# GLP-1 receptor agonist as an effective treatment for breast cancer-related lymphedema: a case report

**DOI:** 10.3389/fonc.2024.1392375

**Published:** 2024-04-18

**Authors:** Fionnuala Crowley, Stav Brown, Emily J. Gallagher, Joseph H. Dayan

**Affiliations:** ^1^ Division of Hematology and Medical Oncology, Department of Medicine, Icahn School of Medicine at Mount Sinai, New York, NY, United States; ^2^ Plastic and Reconstructive Surgery Division, Department of Surgery, Memorial Sloan Kettering Cancer Center, New York, NY, United States; ^3^ Division of Endocrinology, Diabetes and Bone Diseases, Department of Medicine, Icahn School of Medicine at Mount Sinai, New York, NY, United States

**Keywords:** lymphedema, cancer-related lymphedema, glucagon-like peptide 1 receptor agonists, GLP-1, GLP-1RA, breast cancer, case report

## Abstract

**Introduction:**

Lymphedema is a major public health issue for many women undergoing breast cancer treatment. Although weight loss has been reported to be beneficial in the treatment of lymphedema, no studies to date have examined the use of GLP-1RAs for the treatment of secondary lymphedema. This case report describes a patient who experienced significant resolution of her breast cancer-related lymphedema after initiation of a GLP-1RA for weight loss.

**Main symptoms and/or important clinical findings:**

Nine months postoperatively the patient developed arm swelling and disability. While on adjuvant chemo and hormonal therapy, her weight increased dramatically and peaked 4 years later. Corresponding to her weight gain was significant worsening of her symptoms.

**The main diagnoses, therapeutic interventions, and outcomes:**

Due to adjuvant cancer-related weight gain and inability to lose weight with diet and exercise, she was referred for evaluation and diagnosed with lymphedema. The patient started treatment with a Glucagon-like peptide 1 receptor agonist and lost 24% of her body weight over the next 13 months. The improvement in her lymphedema mirrored her weight loss. Her limb volume difference dropped from 10.3% down to 3.4% and she no longer required a compression garment. Her imaging demonstrated return of lymphatic pumping and she experienced a significant improvement in quality of life, assessed by a validated lymphedema-specific patient reported outcome (PROM). She remains on hormonal therapy, no longer needs compression and is back to regular exercise without impairment.

**Conclusions:**

GLP-1 RAs provide a potential medical option for many patients struggling with weight gain and lymphedema. We have observed by all objective measures a significant reduction in lymphedema and the elimination of compression in the case presented as a direct result of GLP-1 RA. This may also reduce a patient’s BMI to the point where they become a good candidate for lymphovenous bypass or vascularized lymph node transplant when indicated.

## Introduction

Lymphedema is a major public health issue for many women undergoing breast cancer treatment. One in three women undergoing axillary lymph node dissection and radiotherapy will develop this incurable and disabling disease (1–3). Swelling, cellulitis, and disability are typical features of this condition. The standard treatment is lifelong compression and physiotherapy, but even the most compliant patients often experience disease progression. If the results of conservative management are limited, surgery is a worthwhile consideration in the appropriate patient with reported efficacy in the literature (4–8). However, not every patient is a surgical candidate. Currently there is no medical therapy that has been widely adopted for lymphedema and there is no cure.

As far back as 1957 studies have shown that obesity can increase the risk of secondary lymphedema (9, 10). This is particularly relevant for patients undergoing breast cancer treatment where chemotherapy and endocrine therapies often result in significant weight gain (11–13). Consequently, a significant increase in body mass index (BMI) can induce or exacerbate lymphedema. Patients frequently struggle with both weight gain and lymphedema along with metabolic derangements secondary to breast cancer therapies.

Glucagon-like peptide 1 receptor agonists (GLP-1RAs) were originally brought to market as treatments for diabetes, and were noted to induce weight loss (14). In more recent times liraglutide, semaglutide and tirzepatide have been approved for chronic weight management in adults with obesity or who are overweight with at least one weight-related condition (such as high blood pressure, type 2 diabetes, or high cholesterol) (15).

Although weight loss has been reported to be beneficial in the treatment of lymphedema, no studies to date have examined the use of GLP-1RAs for the treatment of secondary lymphedema. This case report describes a patient who experienced significant resolution of her breast cancer-related lymphedema after initiation of a GLP-1RA for weight loss.

## Case

This is a 44-year-old woman who presented in 2017 for evaluation of breast cancer-related lymphedema due to arm swelling. She had a history of poorly differentiated stage IIA invasive ductal breast carcinoma with right axillary lymph node involvement. The tumor was estrogen receptor positive and human epidermal growth factor receptor 2 positive. The patient underwent neoadjuvant chemotherapy, followed by right mastectomy and axillary lymph node dissection and adjuvant radiation therapy, chemotherapy and hormonal therapy.

Nine months postoperatively this patient developed mild upper extremity lymphedema—classified as stage 1 based on the International Society of Lymphology (ISL) staging system. Her lymphedema was well controlled but required daily compression with a negligible limb volume difference of 1.7%. She had only mildly impaired quality of life reflected by a low impairment score of 19.1 on the validated Lymphedema Life Impact Scale (LLIS). Indocyanine green lymphangiography (ICG) demonstrated mild abnormal collateralization of lymphatic vessels and presence of lymphatic pumping and flow into the axilla. Her initial weight was 49.9 kg (BMI 19.2 kg/m^2^). While on adjuvant chemo and hormonal therapy, her weight increased dramatically and peaked 4 years later to 66.3 kg (16.4 kg weight gain) and a BMI of 24.9. The This weight gain resulted in significant exacerbation of her lymphedema. Her limb volume difference spiked to 10.3% and her LLIS demonstrated severe disability with an impairment score that nearly tripled to 52.9. Repeat ICG lymphangiography demonstrated severe dermal backflow and lymphatic congestion now without any lymphatic pumping and no flow into the axilla. Her clinical lymphedema stage progressed from ISL 1 (mild lymphedema) to ISL 2 (moderate lymphedema).

**Figure 1:**
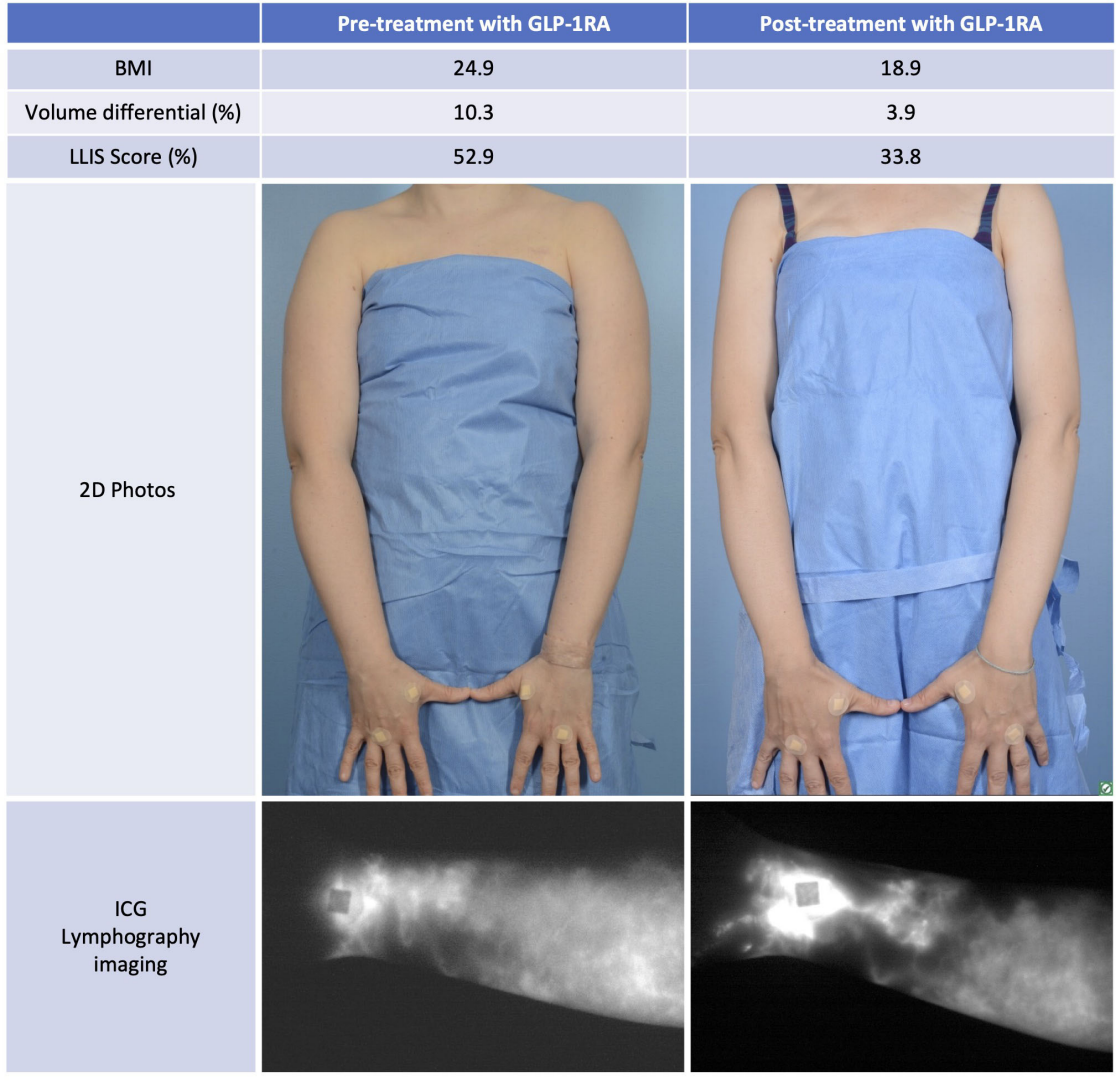
This is a 44 year-old woman with breast cancer-related lymphedema who was treated with semaglutide resulting in significantly reduced limb volume, improved patient reported outcome, and improved ICG lymphangiography. Post-treatment photo is shown at 13 months follow-up without compression.

**Figure 2:**
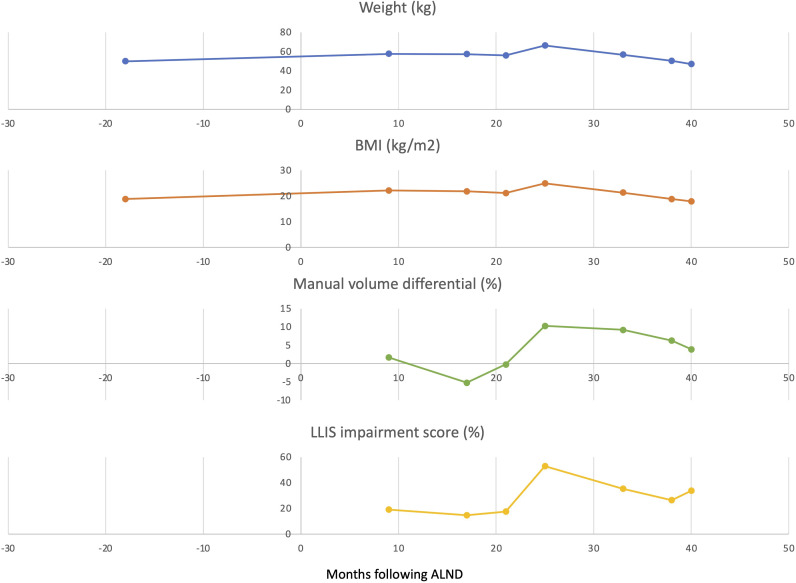
Weight, BMI, impairment score, and limb volume differential scores before and after treatment with GLP-1 RA. Arrows indicate the initiation of GLP-1 RA treatment.

Due to adjuvant cancer-related weight gain and inability to lose weight with diet and exercise, she was referred to endocrinology for evaluation. She was initially started on liraglutide with limited weight response. She was then switched to semaglutide titrated to 1.7 mg weekly and lost 24% of her body weight over the next 13 months. Her weight settled in at 50.3 kg (BMI 18.8). The improvement in her lymphedema mirrored her weight loss. Her limb volume difference dropped from 10.3% down to 3.4% and she no longer required a compression garment. Her LLIS impairment score dropped to 26.5, half of what it was at her peak weight. Follow-up ICG demonstrated return of lymphatic pumping (**Figures 1**, **2**). Her ISL stage reverted to stage 1. She no longer requires compression and is back to regular exercise without impairment or swelling at 30 months following treatment with semaglutide. There were no adverse events observed.

## Discussion

This case report describes a new potential medical treatment for lymphedema using GLP-1RAs. We had followed this patient closely with all available metrics that objectively demonstrated not only resolution of her lymphedema but also eliminated the need for compression. This was a particularly exciting finding as she is similar to many women we see with breast cancer-related lymphedema: node positive disease requiring multimodal adjuvant therapy resulting in significant weight gain leading to or exacerbating their lymphedema. Currently there are no validated medical treatments for lymphedema that have gained traction. The potential for a drug that may help many of our patients is intriguing and worthy of a formal study.

Weight gain is a common consequence of adjuvant treatment for breast cancer (11, 13, 16, 17). There is also a clear association between elevated BMI and lymphedema (9, 10, 18–24). However, the effects of weight loss on lymphedema have yielded mixed results albeit with several confounding factors (20, 25–28). Two publications by Shaw et al, demonstrated modest weight loss led to a significant reduction in limb volume (26, 27). Schmidt and colleagues did not observe an effect of weight loss on limb volume (28). However, the mean weight loss in the experimental groups were 7% to 8% loss in body weight. This may not be an adequate amount of weight loss to register a difference. In contrast, this case report observed a 24% loss in body weight. Finally, limb volume recordings are confounded by any changes in compression during the study period which could affect the volume. The most compelling evidence of a real effect of GLP-1RA in this case report is that this patient no longer required compression and her limb volume was significantly reduced. In contrast, the post-treatment compression requirements in other studies are not reported making it difficult to draw meaningful conclusions.

In this report, both the patient’s impairment score and limb volume correlated with both weight gain and weight loss. She had gained 16.4 kg (36 lbs.), causing a dramatic progression of her lymphedema: her impairment score nearly tripled, and her limb volume increased from 1.7% to greater than 10% despite compression and physiotherapy. It is worthwhile noting that this patient was not obese, nor was she diabetic. After treatment with a GLP-1RA, she had lost 24% of her body weight corresponding to a greater than 30-pound weight loss. This is far more significant weight loss than reported in prior studies which may explain the larger effect on her lymphedema.

The patient in this report is similar to many patients undergoing axillary dissection, radiation, chemotherapy and hormonal therapy. The consequence of lymphedema exacerbated by weight gain from cancer treatment can be debilitating. Weight gain of this nature is often refractory to diet and exercise leaving many patients without the hope of improvement. While we routinely perform lymphatic surgery for lymphedema, many patients are not candidates for surgery because of high BMI or do not want surgical intervention. The GLP-1RAs reliably result in an average weight loss of 15% of the patient’s body weight. The newly improved glucagon-dependent insulinotropic polypeptide/GLP-1 RAs result in more than 20% weight loss in a recent clinical trial (15, 29, 30).

GLP-1RAs may be appropriate in patients with a BMI of 25 or greater and does require a rigorous prospective study to further characterize the role of GLP-1RAs in the treatment of lymphedema. It is also worth investigating pathways that GLP-1RAs may affect in the pathophysiology of lymphedema as it is unclear whether their potential effects are related to weight loss alone or interactions with known inflammatory pathways of lymphedema.

Regarding the mechanism of action, is the GLP-1RA reducing lymphedema because of the induced weight loss or is there another mechanism at play? GLP-1 receptors are widely distributed throughout the body and have multiple biological effects: suppressing appetite, reducing neuroinflammation, regulating blood lipid metabolism and reducing fat deposition (31). GLP-1 RAs also elevate adiponectin levels and reduce leptin levels which may have a direct effect on lymphedema (32, 33). Adiponectin is an adipocyte complement-related protein that exhibits anti-inflammatory properties and is inversely correlated with BMI (34–38). Leptin has pro-inflammatory properties and upregulates the secretion of inflammatory cytokines (39). Adiponectin has been implicated in adipogenesis commonly observed in patients with lymphedema (40). Lymphedema fluid collected from lymphedema patients demonstrate lower adiponectin levels and higher leptin levels compared to their own plasma (35). Interestingly, systemic administration of adiponectin in a lymphedema mouse model effectively reduced lymphedema by inducing lymphatic vessel formation (41). Given the effects of GLP-1 RAs on adiponectin and leptin, there may be beneficial effects on lymphatic function and regeneration but this is a subject for future study.

Lymphedema is a fundamentally immunologic disease characterized by lymph stasis inducing CD4+ T cell activation and T-helper 2 cell differentiation (42, 43). This results in upregulation of chemokines inducing migration of lymphocytes into the skin (44). GLP-1RAs have anti-inflammatory properties that may modulate the immune-system (45, 46). An *in vitro* study by Lieberman et al, demonstrated that GLP-1 RA inhibits chemokine-related migration of human CD4+ lymphocytes (47). The effect of GLP-1RAs on lymphedema’s immunological landscape has not yet been studied but it is possible that GLP-1RAs anti-inflammatory properties may mitigate symptoms of lymphedema. Specific studies assessing the potential immunologic effects of GLP-1 RAs on lymphedema are needed.

It is important to note that the Semaglutide Treatment Effect in People with Obesity (STEP1) clinical trial excluded individuals with a history of malignant neoplasms within five years of screening and therefore, there are no data from clinical trials regarding the weight loss effects, and potential risks or benefits of semaglutide in individuals with breast cancer. Numerous studies have found a positive correlation between obesity and breast cancer incidence, recurrence, and mortality, particularly in postmenopausal patients with estrogen receptor-positive breast cancer, and pre-menopausal triple negative breast cancer (TNBC) (48). Weight loss would therefore theoretically be beneficial to reduce these risks. Surprisingly, a systematic review and meta-analysis of randomized controlled trials found no difference in the risk of developing breast cancer in individuals taking GLP-1RAs compared with controls (49). The effects of GLP-1RAs on breast cancer progression has led to conflicting findings in *in vitro* and preclinical rodent models. In the human estrogen receptor-positive MCF7 breast cancer cell line, GLP-1RA have consistently been reported to have anti-proliferative and pro-apoptotic effects (50–53). In contrast, in the MDA-MB-231 TNBC cell line, the GLP1-RA liraglutide was found to stimulate cell growth *in vitro*, but exendin-4 reduced the proliferation (53–55). *In vivo*, liraglutide was also reported to accelerate the growth of murine 4T1 cells in a syngeneic model (55), but exendin-4 inhibited the growth of MDA-MB-231 and MDA-MB-468 TNBC xenografts (53). Therefore, the preclinical studies support a direct anti-tumor effect of GLP-1RA in hormone receptor positive breast cancers, the subtype of breast cancer with which our patient was being treated; however, more research is needed to understand the conflicting effects of these medications in other subtypes of breast cancer.

## Conclusion

The possibility that a GLP-1RA may significantly improve lymphedema in patients with excess weight gain is an exciting one. Weight gain is a common consequence of breast cancer treatment often exacerbating lymphedema. GLP-1 RAs provide a potential medical option for many patients struggling with weight gain and lymphedema. We have observed by all objective measures a significant reduction in lymphedema and the elimination of compression in the case presented as a direct result of GLP-1 RA treatment. This may also reduce a patient’s BMI to the point where they become a good candidate for lymphovenous bypass or vascularized lymph node transplant when indicated. At this time, however, these conclusions are limited to speculation given the nature of a single case report. Future prospective studies are needed to quantify the efficacy and better understanding the cellular mechanisms of GLP-1 RAs on lymphedema.

## Patient perspective

Following the patient’s weight gain induced by adjuvant therapy, she reported significant limb swelling that required daily use of compression garments and resulted in a substantial quality of life impairment. Following 13 months of treatment with semaglutide, she reported significant improvement in her limb volume, stopped wearing a compression garment entirely and experienced a significant quality of life improvement.

## Data availability statement

The original contributions presented in the study are included in the article/supplementary material. Further inquiries can be directed to the corresponding author.

## Ethics statement

Ethical approval was not required for the study involving humans in accordance with the local legislation and institutional requirements. Written informed consent to participate in this study was not required from the participants or the participants’ legal guardians/next of kin in accordance with the national legislation and the institutional requirements. Written informed consent was obtained from the individual(s) for the publication of any potentially identifiable images or data included in this article.

## Author contributions

FC: Conceptualization, Data curation, Writing – original draft, Writing – review & editing. SB: Data curation, Writing – original draft, Writing – review & editing, Conceptualization. EG: Conceptualization, Data curation, Writing – original draft, Writing – review & editing. JD: Conceptualization, Data curation, Supervision, Writing – original draft, Writing – review & editing.
